# SNX8 modulates innate immune response to DNA virus by mediating trafficking and activation of MITA

**DOI:** 10.1371/journal.ppat.1007336

**Published:** 2018-10-15

**Authors:** Jin Wei, Huan Lian, Wei Guo, Yun-Da Chen, Xia-Nan Zhang, Ru Zang, Li Zhong, Qing Yang, Ming-Ming Hu, Wei-Wei Luo, Hong-Bing Shu, Shu Li

**Affiliations:** 1 Medical Research Institute, School of Medicine, Wuhan University, Wuhan, China; 2 College of Life Sciences, Wuhan University, Wuhan, China; 3 Wuhan Institute of Virology, Chinese Academy of Sciences, Wuhan, China; University of Southern California, UNITED STATES

## Abstract

MITA (also called STING) is a central adaptor protein in innate immune response to cytosolic DNA. Cellular trafficking of MITA from the ER to perinuclear microsomes after DNA virus infection is critical for MITA activation and onset of innate antiviral response. Here we found that SNX8 is a component of DNA-triggered induction of downstream effector genes and innate immune response. *Snx8*^*-*/-^ mice infected with the DNA virus HSV-1 exhibited lower serum cytokine levels and higher viral titers in the brains, resulting in higher lethality. Mechanistically, SNX8 recruited the class III phosphatylinositol 3-kinase VPS34 to MITA, which is required for trafficking of MITA from the ER to perinuclear microsomes. Our findings suggest that SNX8 is a critical component in innate immune response to cytosolic DNA and DNA virus.

## Author summary

Infection by virus, such as the DNA virus herpes simplex virus 1, induces the host cells to express proteins that mediate antiviral immune response. The protein called MITA plays an essential role in the process of antiviral immune response. After viral infection, MITA traffics from the endoplasmic reticulum (ER) to perinuclear punctuate structures, a process required for its activation and induction of downstream antiviral genes. How the trafficking of MITA is regulated remains enigmatic. In this study, we found that a protein called SNX8 plays a critical role in regulation of MITA trafficking from the ER to perinuclear punctuate structures. SNX8 does this by recruiting the VPS34-containing translocon machinery to MITA. Deficiency of SNX8 impaired antiviral immune response and rendered the mice more sensitive to DNA virus-induced death. Our results suggest that SNX8 is a critical component in antiviral immune response to DNA virus.

## Introduction

Innate immune response is pivotal for host defense against microbial pathogens. Cytosolic DNA derived from invading pathogens triggers signaling events that lead to induction of downstream anti-microbial effector genes [1–3]. It has been demonstrated that the nucleotidyltransferase family member cyclic GMP-AMP (cGAMP) synthase (cGAS) is a ubiquitously expressed sensor of cytosolic DNA in various cell types [4,5]. Upon recognition of cytosolic DNA, cGAS utilizes ATP and GTP as substrates to catalyze the synthesis of the second messenger cGAMP, which binds to and activates the endoplasmic reticulum (ER)-located central adaptor protein MITA (also called STING, MPYS, ERIS, or TMEM173) [6–9]. After binding to cGAMP, MITA traffics from the ER via Golgi apparatus to perinuclear microsomes, and in these processes TBK1 and IRF3 are recruited to MITA. In the MITA-associated complex, TBK1 firstly phosphorylates MITA at Ser366 and then phosphorylates IRF3, leading to activation of IRF3 and induction of downstream effector genes such as type I interferons (IFNs) and proinflammatory cytokines [5,10–12].

The trafficking of MITA is critical for its activity in response to cytosolic DNA [13]. It has been demonstrated that the TRAPβ, Sec61β and Sec5 containing translocon complex as well as the class III phosphatylinositol 3-kinase (PI3K) VPS34 play critical roles for the trafficking and activation of MITA [7,12,14,15]. However, the regulatory mechanisms for MITA trafficking are still not well understood.

Sorting nexin 8 (SNX8) belongs to the sorting nexin protein family, which is involved in endocytosis and endosomal sorting [16,17]. Recently, we have demonstrated that SNX8 is a component of IFNγ-triggered noncanonical signaling pathway [16,17]. In this report, we identified SNX8 as a component of DNA-triggered innate immune response. Deficiency of SNX8 inhibited DNA- or DNA virus-triggered induction of downstream antiviral genes and innate antiviral response. Biochemical analysis indicated that SNX8 links VPS34 to MITA, which is required for trafficking and activation of MITA. Our findings reveal that SNX8 is a critical component in innate immune response to cytosolic DNA and DNA virus.

## Results

### SNX8 positively regulates DNA virus-triggered signaling

**Fig 1:**
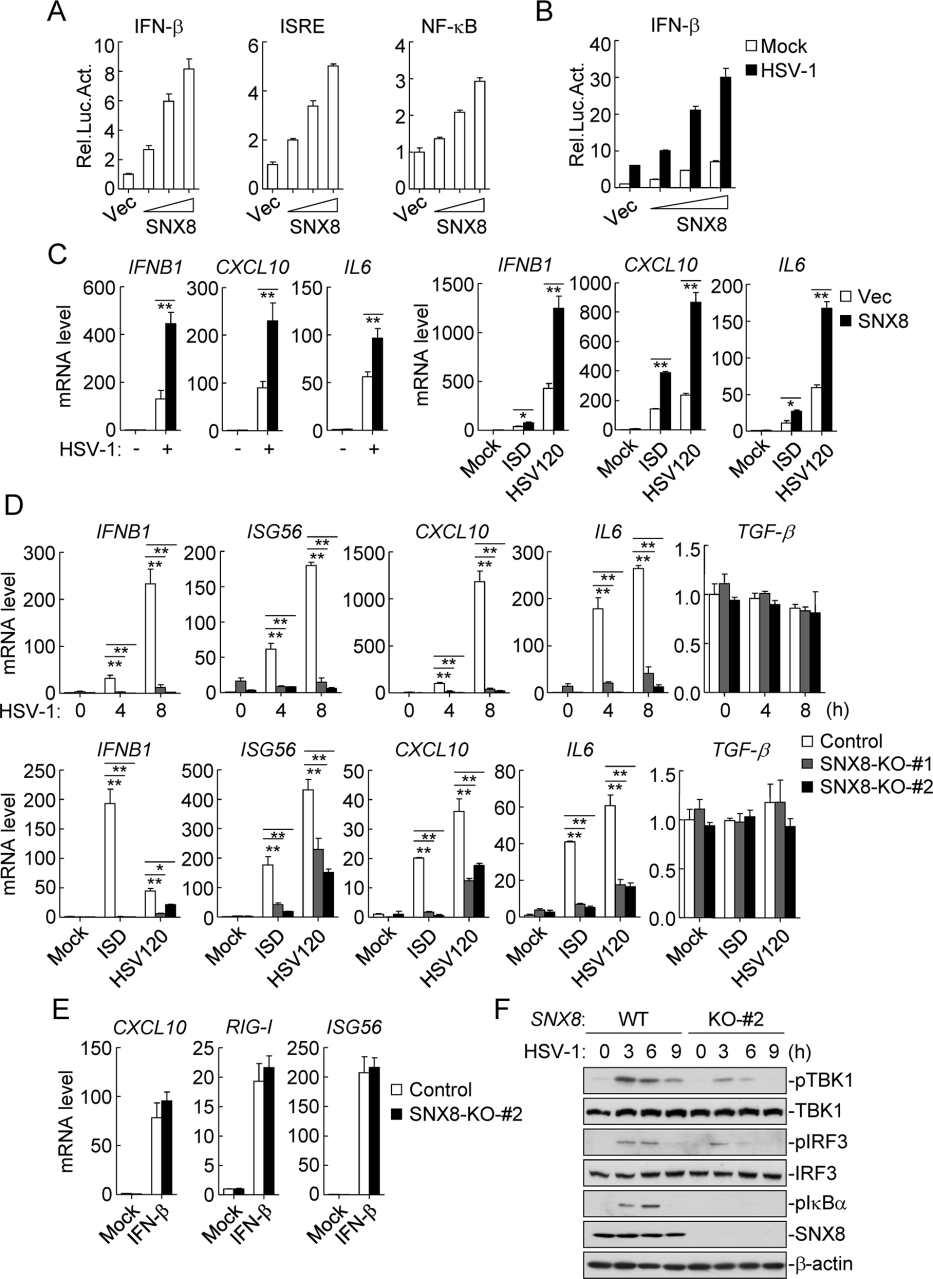
SNX8 positively regulates DNA virus-triggered signaling. **(A)** HeLa cells (1x10^5^) were transfected with IFN-β (0.05 μg), ISRE (0.05 μg) and NF-κB (5 ng) reporter plasmids and increased amounts of SNX8 (0.05, 0.1, 0.2 μg) for 24 h before luciferase assays were performed. (B) HeLa cells (1x10^5^) were transfected with the IFN-β reporter (0.05 μg) plasmids and increased amounts of SNX8 (0.05, 0.1, 0.2 μg). Twenty hours after transfection, cells were left un-infected or infected with HSV-1 (MOI = 1) for 10 h before luciferase assays were performed. (C) SNX8 stable-expressing HFFs (4x10^5^) were infected with HSV-1 (MOI = 1) or transfected with ISD and HSV120 (3 μg/ml) for 6 h before qPCR analysis. (D) SNX8-deficient HFFs (4x10^5^) were infected with HSV-1 (MOI = 1) or transfected with ISD or HSV120 (3 μg/ml) for 6 h before qPCR analysis. (E) SNX8-deficient HFFs (4x10^5^) were left un-treated or treated with IFN-β (100 ng/ml) for 6 h before qPCR analysis. (F) SNX8-deficient HFFs (4x10^5^) were left un-infected or infected with HSV-1 (MOI = 1) for the indicated times followed by immunoblot analysis. (*p<0.05, **p<0.01).

Our previous study has demonstrated that SNX8 mediates IFNγ-triggered non-canonical signaling pathway and host defense against Listeria infection [16]. In this study, we investigated the role of SNX8 in innate antiviral response. In reporter assays, overexpression of SNX8 dose-dependently activated the IFN-β promoter, which requires coordinative and cooperative activation of the transcription factors IRF3 and NF-κB. Consistently, overexpression of SNX8 activated ISRE (an enhancer motif for IRF3) and NF-κB in a dose-dependent manner in reporter assays (Fig 1A). Further experiments indicated that SNX8 potentiated HSV-1-triggered activation of the IFN-β promoter in a dose-dependent manner in HeLa cells (Fig 1B). Overexpression of SNX8 also potentiated the transcription of *IFNB1*, *CXCL10* and *IL6* genes induced by HSV-1 and transfected synthetic DNAs ISD (IFN-stimulating DNA) and HSV120 (DNA of 120 basepairs representing the genomes of HSV-1) (Fig 1C). These results suggest that SNX8 is involved in DNA virus-triggered induction of downstream antiviral genes.

To investigate the role of endogenous SNX8 in DNA virus-triggered signaling, we constructed SNX8-deficient human foreskin fibroblasts (HFFs) by the CRISPR-Cas9 method. We found that transcription of *IFNB1*, *ISG56*, *CXCL10* and *IL6* genes induced by HSV-1 and transfected ISD and HSV120 was dramatically inhibited in SNX8-deficient HFFs in comparison to wild-type cells (Fig 1D). In contrast, the transcription of *TGF-β* following HSV-1 infection and transfection of cytosolic dsDNA was comparable between SNX8-deficient and control HFFs (Fig 1D). In similar experiments, SNX8-deficiency did not affect IFN-β-triggered induction of downstream genes such as *CXCL10*, *RIG-I*, and *ISG56* (Fig 1E). Consistently, HSV-1-induced phosphorylation of TBK1, IRF3 and IκBα, which are hallmarks of activation of the DNA-triggered signaling pathways, were markedly inhibited in SNX8-deficient cells (Fig 1F). These results suggest that SNX8 is essential for DNA virus-triggered induction of downstream genes.

### Snx8 is essential for innate immune response to DNA virus

**Fig 2:**
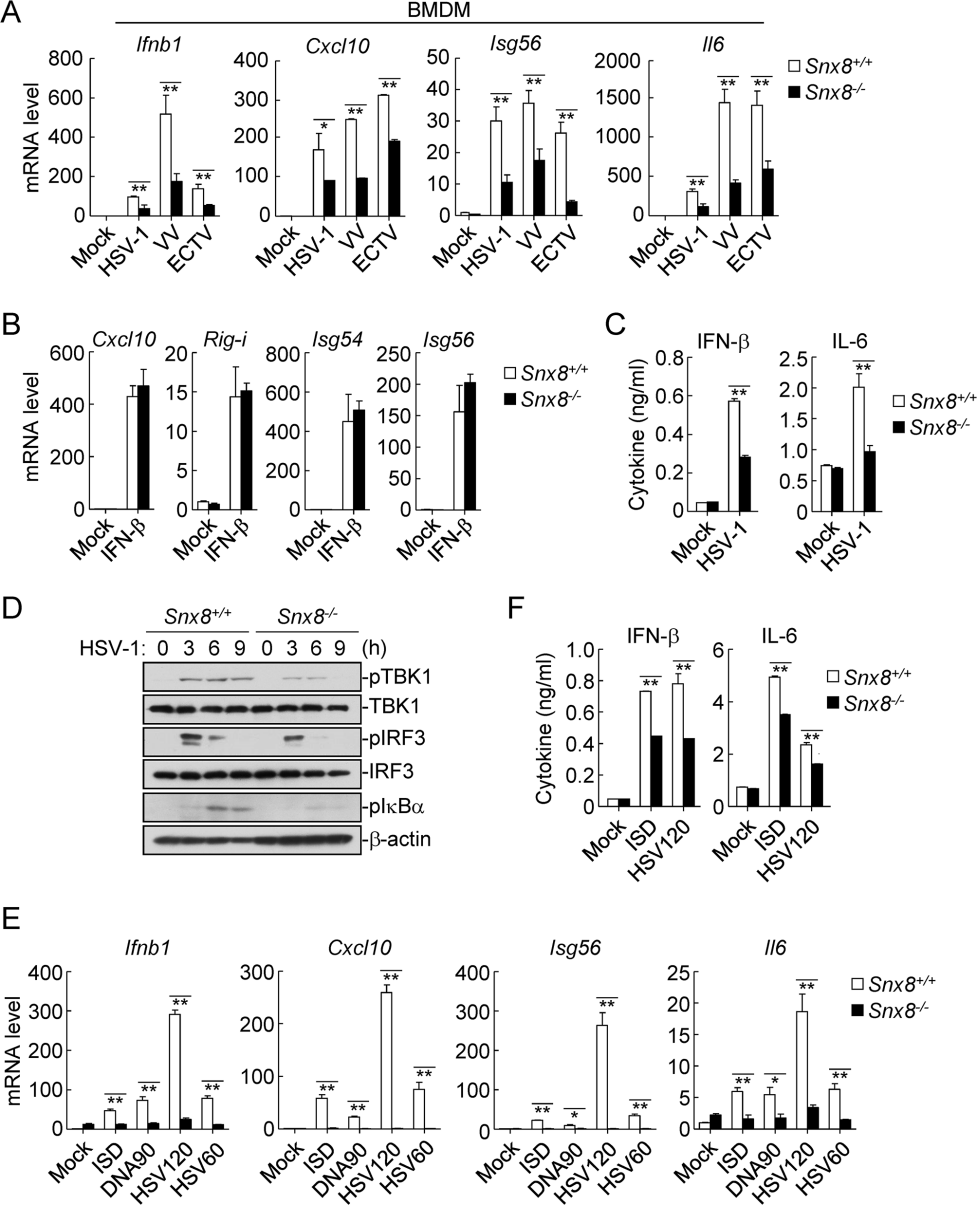
SNX8 is essential for DNA virus-triggered signaling. (A) *Snx8*^*+/+*^ and *Snx8*^*-/-*^ BMDMs (4x10^5^) were left un-infected or infected with HSV-1, VV, or ECTV (MOI = 1) for 6 h before qPCR analysis. (B*) Snx8*^*+/+*^ and *Snx8*^*-/-*^ BMDMs (4x10^5^) were left un-treated or treated with IFN-β (100 ng/ml) for 6 h before qPCR analysis. (C) *Snx8*^*+/+*^ and *Snx8*^*-/-*^ BMDMs (4x10^5^) were left un-infected or infected with HSV-1 (MOI = 1) for 18 h. The culture media were collected for ELISA. (D) *Snx8*^*+/+*^ and *Snx8*^*-/-*^ BMDMs (4x10^5^) were infected with HSV-1 (MOI = 1) for the indicated times before immunoblot analysis. (E) *Snx8*^*+/+*^ and *Snx8*^*-/-*^ BMDMs (4x10^5^) were transfected with the indicated nucleic acids (3 μg/ml) for 3 h before qPCR analysis. (F) *Snx8*^*+/+*^ and *Snx8*^*-/-*^ BMDMs (4x10^5^) were transfected with the indicated nucleic acids (3 μg/ml) for 18 h. The culture media were collected for ELISA. (*p<0.05, **p<0.01).

We next investigated innate immune response to DNA virus in primary cells from *Snx8*^*+/+*^ and *Snx8*^*-/-*^ mice. We found that induction of *Ifnb1*, *Isg56*, *Cxcl10* and *Il6* genes were significantly inhibited in *Snx8*^*-/-*^ bone marrow-derived macrophages (BMDMs) and mouse lung fibroblasts (MLFs) in comparison with their wild type counterparts after infection with three different types of DNA viruses, including HSV-1, vaccinia virus (VV), and ectromelia virus (ECTV) (Fig 2A and S1A Fig). In similar experiments, the mRNA levels of *Cxcl10*, *Rig-I*, *Isg54* and *Isg56* induced by IFN-β was comparable between *Snx8*^*-/-*^ and *Snx8*^*+/+*^ BMDMs (Fig 2B). In addition, the secretion of IFN-β and IL-6 cytokines were impaired in *Snx8*^*-/-*^ BMDMs following HSV-1 infection (Fig 2C). Consistently, HSV-1-induced phosphorylation of TBK1, IRF3 and IκBα was markedly inhibited in *Snx8*^*-/-*^ BMDMs and MLFs (Fig 2D and S1B Fig). In addition, transcription of *Ifnb1*, *Cxcl10*, *Isg56* and *Il6* genes induced upon transfection of DNAs including ISD, DNA90 (dsDNA of approximately 90 basepairs), HSV60 and HSV120 (dsDNA 60- or 120-mers representing the genomes of HSV-1) was impaired in *Snx8*^*-/-*^ BMDMs and MLFs (Fig 2E and S1C Fig). Consistently, secretion of IFN-β and IL-6 cytokines induced by transfected ISD and HSV120 was significantly inhibited in *Snx8*^*-/-*^ BMDMs (Fig 2F). Collectively, these results suggest that SNX8 is essential for induction of downstream antiviral genes following DNA virus infection or cytosolic DNA stimulation in primary murine cells.

**Fig 3:**
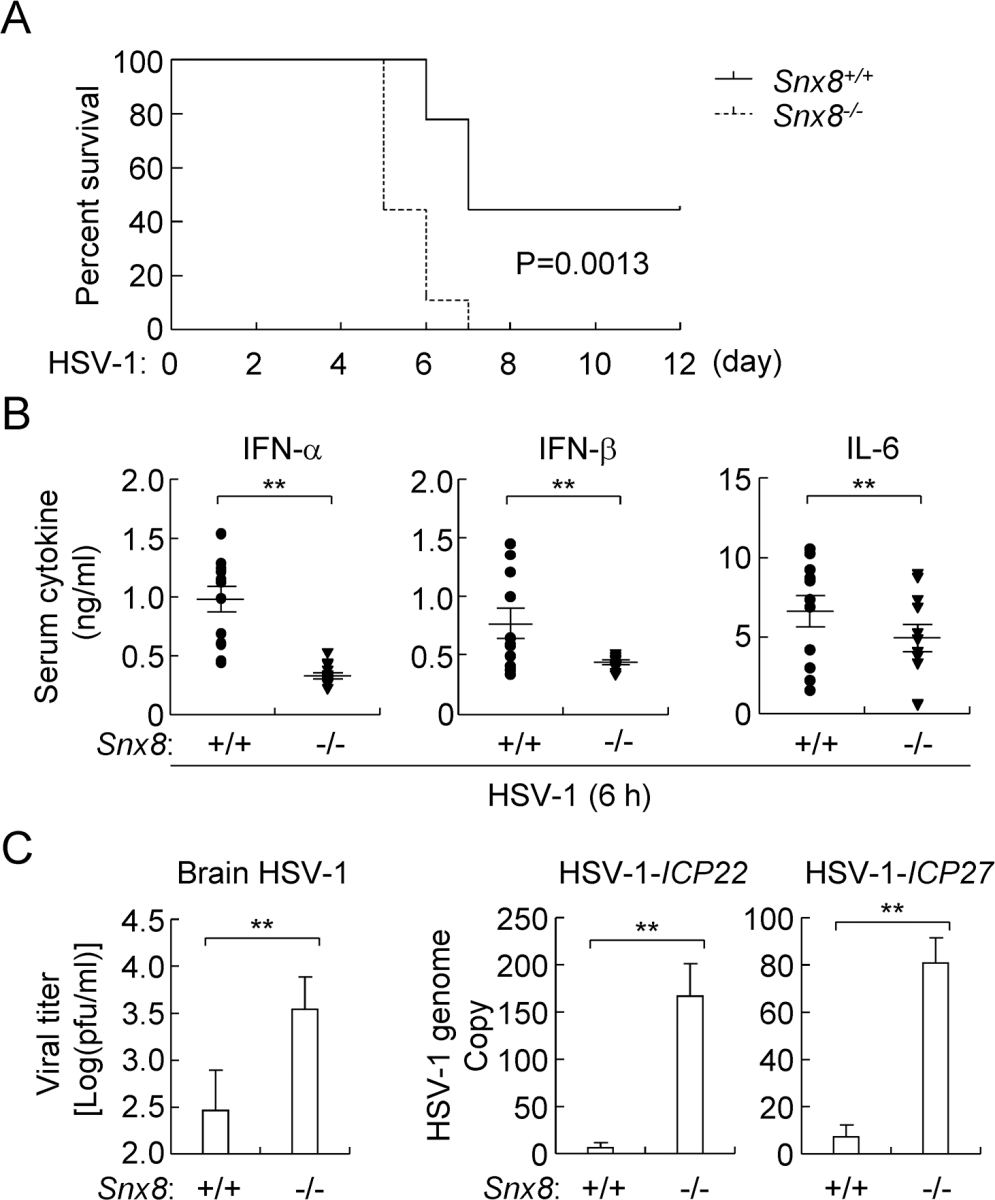
SNX8 is essential for host defense against HSV-1 infection in mice. (A) *Snx8*^*+/+*^ and *Snx8*^*-/-*^ mice (n = 10) were infected intraperitoneally (i.p.) with HSV-1 at 1.5 × 10^7^ pfu per mouse, and the survivals of mice were recorded daily for 12 days. (B) *Snx8*^*+/+*^ and *Snx8*^*-/-*^ mice (n = 13) were infected intraperitoneally (i.p.) with HSV-1 at 1.5 × 10^7^ pfu per mouse for 6 h before serum cytokines were measured by ELISA. (**p<0.01). (C) *Snx8*^*+/+*^ and *Snx8*^*-/-*^ mice (n = 3) were infected intraperitoneally (i.p.) with HSV-1 at 1 × 10^7^ pfu per mouse, and the brains were retrieved 6 days later for viral load measurement (left histograph) and q-PCR analysis of HSV-1 genomic copies (right histograph). (**p<0.01).

To investigate the roles of SNX8 in host defense against viral infection *in vivo*, we infected *Snx8*^*+/+*^ and *Snx8*^*-/-*^ mice with HSV-1 by intraperitoneal (i.p.) injection and monitored their survival. The results indicated that *Snx8*^*-/-*^ mice were more susceptibility to HSV-1-induced death than their wild-type littermates (Fig 3A). In addition, the serum cytokines induced by HSV-1 infection, including IFN-α, IFN-β and IL-6, were severely impaired in *Snx8*^*-/-*^ in comparison to their wild-type littermates (Fig 3B). Moreover, the brain tissues of *Snx8*^*-/-*^ mice infected with HSV-1 (a neurotropic DNA virus) for 6 days showed higher HSV-1 viral loads and genomic DNA copies in comparison to those of infected wild-type littermates (Fig 3C). Collectively, these data suggest that SNX8 is essential for host defense against HSV-1 infection in mice.

### SNX8 is associated with MITA

**Fig 4:**
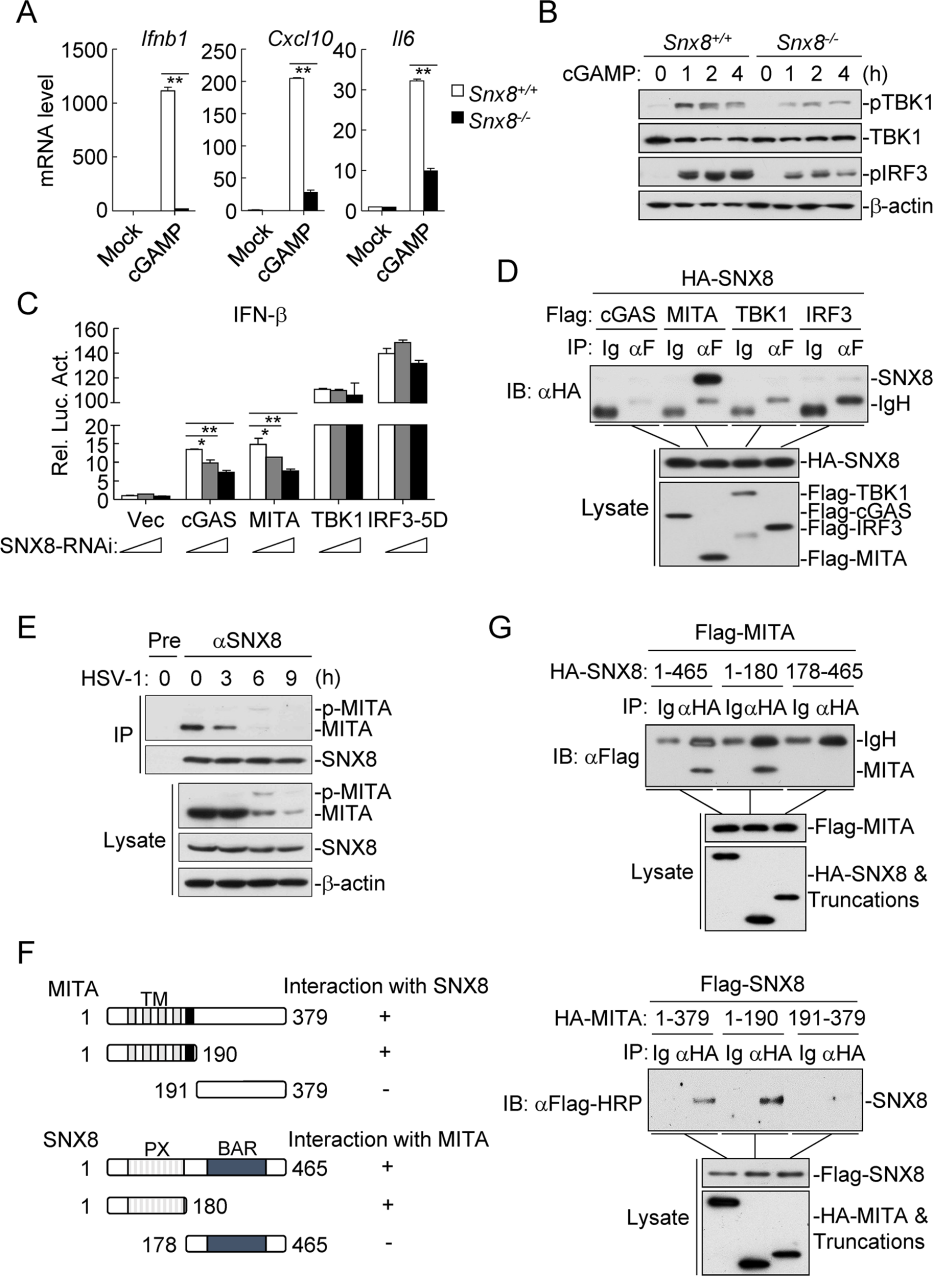
SNX8 is associated with MITA. **(A)** *Snx8*^*+/+*^ and *Snx8*^*-/-*^ MLFs (4x10^5^) were left un-treated or treated with cGAMP (0.2 μg/ml) for 3 h before qPCR analysis. (B) *Snx8*^*+/+*^ and *Snx8*^*-/-*^ MLFs (4x10^5^) were left un-treated or treated with cGAMP (0.2 μg/ml) for the indicated times before immunoblot analysis. (C) HEK293 cells (1x10^5^) were first transfected with increased amounts of SNX8 RNAi (0.4 μg) for 24 h, and then re-transfected with the IFN-β reporter (0.05 μg) and indicated expression plasmids (0.1 μg each) for 24 h before luciferase assays. (*p<0.05, **p<0.01). (D) HEK293 cells (2x10^6^) were transfected with the indicated plasmids (5 μg each) for 24 h before co-immunoprecipitation and immunoblot analysis were performed. (E) MLFs (3x10^7^) were left un-infected or infected with HSV-1 for the indicated times before endogenous co-immunoprecipitation and immunoblot analysis were performed. (F&G) HEK293 cells (2x10^6^) were transfected with the indicated truncations (5 μg each) before co-immunoprecipitation and immunoblot analysis were performed.

To investigate the mechanisms of SNX8 in DNA virus-triggered signaling, we examined whether SNX8 is involved in the induction of downstream genes triggered by intracellular cGAMP. We found that cGAMP-induced transcription of *Ifnb1*, *Isg56*, *Cxcl10* and *Il6* genes were significantly dampened in *Snx8*^*-/-*^ in comparison with wild-type MLFs (Fig 4A). In addition, cGAMP-induced phosphorylation of TBK1 and IRF3 was also impaired in *Snx8*^*-/-*^ MLFs (Fig 4B). These results suggest that SNX8 acts downstream of cGAMP and upstream of TBK1-IRF3. Consistently, knockdown of SNX8 inhibited cGAS- and MITA- but not TBK1- or IRF3-5D- (a constitutive active mutant of IRF3) mediated activation of the IFN-β promoter in a dose-dependent manner (Fig 4C).

We next determined whether SNX8 is associated with signaling components in DNA virus-triggered pathways. Transient transfection and co-immunoprecipitation experiments indicated that SNX8 was associated with MITA, but not cGAS, TBK1 or IRF3 (Fig 4D). Endogenous co-immunoprecipitation experiments indicated that SNX8 constitutively interacted with MITA in un-infected and early-infected cells, but their association was undetectable at late phase (6–9 h) of HSV-1 infection due to down-regulation of MITA (Fig 4E). Domain mapping analysis indicated that the N-terminal transmembrane domains of MITA (aa1-190) and the N-terminal domain of SNX8 (aa1-180) were required for their association (Fig 4F & 4G). qPCR analysis indicated that reconstitution of SNX8 and SNX8(1–180) but not the MITA binding-defective mutant SNX8(178–465) rescued HSV-1-induced transcription of *Ifnb1* in *Snx8*^*-/-*^ MLFs, suggesting that the interaction of SNX8 with MITA is important for the its role in modulating MITA activation (S1D Fig).

### SNX8 is essential for the trafficking of MITA

**Fig 5:**
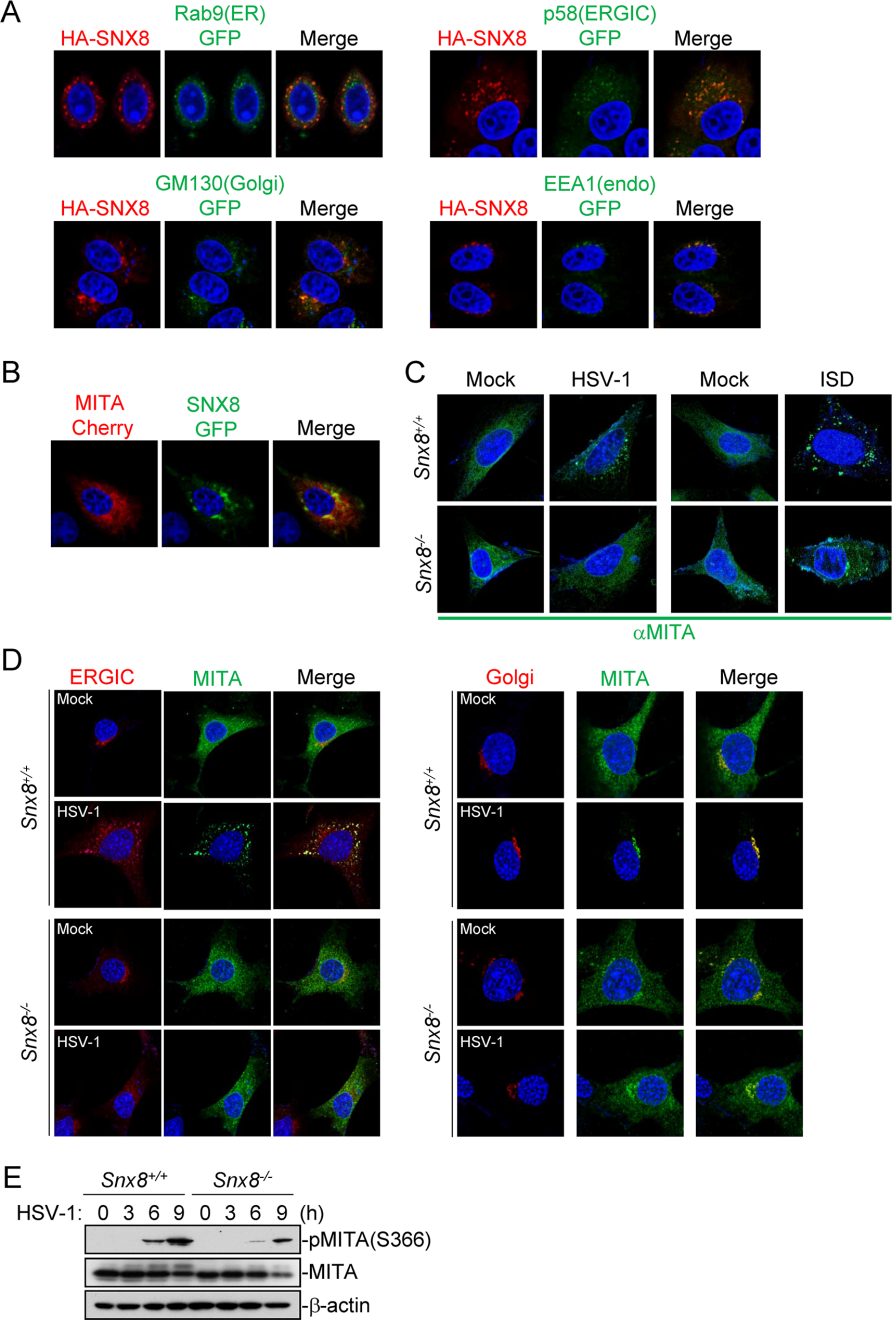
SNX8 facilitates the trafficking and activation of MITA. **(A)** HeLa cells (1x10^5^) were transfected with HA-SNX8 (0.1 μg) and GFP-tagged Rab9 (ER marker) (0.3 μg), p58 (ERGIC marker) (0.3 μg), GM130 (Golgi marker) (0.3 μg), and EEA1 (endosome marker) (0.3 μg) for 20 h before immunostaining was performed with anti-HA. (B) HeLa cells (1x10^5^) were transfected with GFP-SNX8 (0.1 μg) and MITA-Cherry (0.2 μg) for 20 h before confocal microscopy. (C) *Snx8*^*+/+*^ and *Snx8*^*-/-*^ MEFs (1x10^5^) stably transduced with MITA were infected with HSV-1 (MOI = 1) or transfected with ISD (2 μg/ml) for 3 h before immunostaining was performed with the indicated antibodies. (D) *Snx8*^*+/+*^ and *Snx8*^*-/-*^ MLFs (1x10^5^) stably transduced with MITA were infected with HSV-1 (MOI = 1) for 4 h before immunostaining was performed with anti-MITA, anti-p58 (ERGIC) and anti-GM130 (Golgi). (E) Effects of SNX8-deficiency on HSV-1-induced phosphorylation of MITA. *Snx8*^*+/+*^ and *Snx8*^*-/-*^ MLFs (4x10^5^) were left un-infected or infected with HSV-1 (MOI = 1) for the indicated times before immunoblotting analysis.

Cellular trafficking of MITA from the ER via Golgi to perinuclear microsomes is critically involved in its activation. We investigated whether SNX8 is involved in the trafficking of MITA. Confocal microscopy indicated that SNX8 was localized in the ER, ER-Golgi intermediate compartment (ERGIC), Golgi and endosome (Fig 5A), which is similar with the localization of MITA. In addition, we found that SNX8 and MITA co-localized in the cytoplasm (Fig 5B). Notably, HSV-1 infection or transfection of synthetic ISD caused the accumulation of MITA in perinuclear microsomes in *Snx8*^*+/+*^ MLFs, but this accumulation was completely inhibited in *Snx8*^*-/-*^ MLFs (Fig 5C). Furthermore, confocal microscopy revealed that SNX8-deficiency markedly inhibited the trafficking of MITA from the ER via Golgi to perinuclear microsomes induced by infection of HSV-1 (Fig 5D). These data suggest that SNX8 is required for the trafficking of MITA from the ER to perinuclear microsomes.

It has been demonstrated that during its trafficking, MITA is phosphorylated, activated and recruits IRF3 [18]. We found that SNX8-deficiency dramatically inhibited HSV-1-induced phosphorylation of MITA at Ser366 in MLFs (Fig 5E). These date suggest that SNX8 is critical for the trafficking and activation of MITA upon DNA virus infection.

### SNX8 mediates MITA-VPS34 association

**Fig 6:**
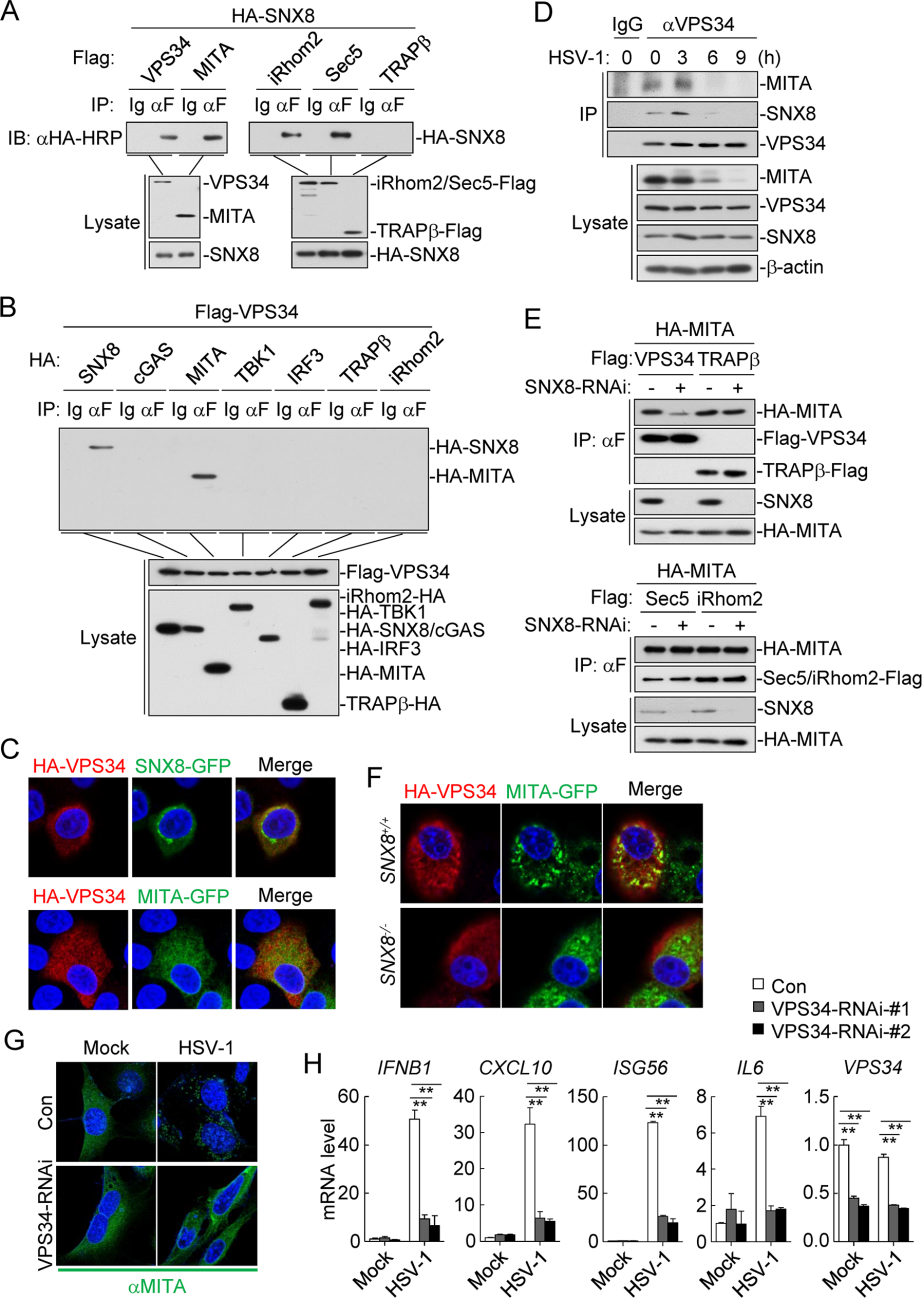
SNX8 is essential for VPS34-mediated trafficking of MITA. **(A)** HEK293 cells (2x10^6^) were transfected with HA-SNX8 and the indicated plasmids (5μg each) for 24 h before co-immunoprecipitation and immunoblot analysis were performed. (B) HEK293 cells (2x10^6^) were transfected with Flag-VPS34 (5 μg) and the indicated plasmids (5 μg each) for 24 h before co-immunoprecipitation and immunoblot analysis were performed. (C) HeLa cells (1x10^5^) were transfected with HA-VPS34 (0.2 μg) and GFP-tagged SNX8 (0.1 μg) and MITA (0.1 μg) for 20 h before immunostaining was performed with anti-HA. (D) MLFs (3x10^7^) were left un-infected or infected with HSV-1 (MOI = 1) for the indicated times before endogenous co-immunoprecipitation and immunoblot analysis were performed. (E) HEK293 cells (2x10^6^) were transfected with the indicated plasmids (5 μg each) for 24h before co-immunoprecipitation and immunoblot analysis were performed. (F) *SNX8*^*+/+*^ and *SNX8*^*-/-*^ HeLa cells (1x10^5^) were transfected with HA-VPS34 (0.2 μg) and GFP-tagged MITA (0.1 μg) for 20 h before immunostaining was performed with anti-HA. (G) MEFs cells (2x10^5^) were transfected with VPS34 siRNA (2 μg) for 36 h and then infected with HSV-1 (MOI = 1) for 3 h. Immunostaining were performed with the indicated antibodies. (H) VPS34-RNAi stable-knockdown THP1 cells (4x10^5^) were infected with HSV-1 (MOI = 1) for 6 h before qPCR analysis. (*p<0.05, **p<0.01).

Several studies have demonstrated that the PI3K VPS34 as well as iRhom2-mediated recruitment of the TRAPβ translocon complex play important roles in the trafficking of MITA [7,12,14,15]. In co-immunoprecipitation experiments, SNX8 interacted with MITA, VPS34, iRhom2, Sec5 but not TRAPβ (Fig 6A). Interestingly, VPS34 interacted with MITA and SNX8 but not TRAPβ (Fig 6B). Consistently, confocal microscopy indicated that VPS34 was co-localized with SNX8 and MITA in the cytoplasm (Fig 6C). Furthermore, endogenous co-immunoprecipitation experiments indicated that VPS34 was associated with MITA and SNX8 in un-infected and early-infected (3 h) but not late-infected (6–9 h) cells, which might be caused by the degradation of MITA in late-infected cells (Fig 6D). Knockdown of SNX8 inhibited the association of MITA with VPS34 but not that of MITA with TRAPβ, Sec5 or iRhom2 (Fig 6E). In addition, confocal microscopy indicated that SNX8-deficiency markedly inhibited the co-localization of VPS34 and MITA in HeLa cells (Fig 6F). These results suggest that SNX8 mediates the MITA-VPS34 but not the MITA-iRhom2-TRAPβ association. Consistently, knockdown of VPS34 inhibited HSV-1-induced trafficking of MITA to microsomes (Fig 6G). In reporter assays, knockdown of VPS34 inhibited MITA- and MITA/SNX8-mediated activation of ISRE (S2A Fig), as well as HSV-1-induced transcription of downstream genes including *IFNB1*, *CXCL10*, *ISG56* and *IL6* (Fig 6H). In addition, VPS34-IN1, a selective inhibitor of VPS34, inhibited HSV-1-induced transcription of downstream genes in a dose-dependent manner (S2B Fig). These results suggest that SNX8-mediated recruitment of VPS34 is essential for the trafficking and activation of MITA.

## Discussion

In this study, we investigated the roles of SNX8 in DNA virus- or cytosolic dsDNA- triggered signaling. SNX8-deficient cells failed to effectively produce IFNs and other cytokines in response to infection with DNA virus and transfected with cytosolic DNA. *Snx8*^*-/-*^ mice exhibited lower serum cytokine levels and higher viral titers in brain, resulting in higher lethality. These findings establish a critical role for SNX8 in innate immune response to cytosolic dsDNA and DNA virus. In our preliminary study, we also found that SNX8 was involved in RNA virus- triggered induction of downstream antiviral genes, which will be reported in a separate study.

Several evidences suggest that SNX8 targets MITA for its regulation of DNA virus-triggered signaling. First, SNX8 deficiency impaired cGAMP-induced signaling, which provided us with the clue that linked SNX8 to MITA pathway. Second, knockdown of SNX8 inhibited cGAS- and MITA-mediated activation of the IFN-β promoter, but not TBK1 or IRF3-5D. Third, SNX8 interacted with MITA in un-infected or early infected with HSV-1. These data suggest that SNX8-mediated DNA virus-triggered signaling is dependent on MITA.

Our results suggest that SNX8 acts as a link for VPS34-mediated trafficking and activation of MITA. The trafficking of MITA from ER to perinuclear microsomes was impaired in *Snx8*^*-/-*^ MEFs infected with HSV-1 or transfected with ISD, suggesting that SNX8 is essential for trafficking of MITA. Co-immunoprecipitation experiments indicated that SNX8 was constitutively associated with VPS34 and MITA. Knockdown of SNX8 impaired the association of VPS34 with MITA. In addition, knockdown of VPS34 inhibited the synergetic effects of SNX8 on MITA-mediated activation of ISRE. These data suggest that SNX8 acts as a link for MITA-VPS34 translocation complex and promotes the trafficking of MITA, which is essential for effective activation of MITA-mediated signaling. In conclusion, our findings uncover previously uncharacterized roles of SNX8 in mediating MITA-dependent innate immune response against DNA virus.

## Materials and methods

### Ethics statement

*Snx8*^*-/-*^ mice on the C57BL/6 background were generated by the CRISPR/Cas9 method and obtained from the Wuhan University A3 Animal Center. All mice were maintained in Specific Pathogen Free facility of Wuhan University College of Life Sciences. The animal care and use protocol was adhered to the Chinese National Laboratory Animal-Guideline for Ethical Review of Animal Welfare. The protocols and procedures for mice experiments in this study were approved by the Wuhan University College of Life Sciences Animal Care and Use Committee guidelines (approval number WDSKY0200902-2).

### Reagents, antibodies, cells and viruses

3′3′-cGAMP, Lipofectamine 2000 (InvivoGen); poly(dA:dT), DNA90, ISD, HSV60, HSV120 (Sangon Biotech); polybrene (Millipore); SYBR (Bio-Rad); FuGene and Dual-Specific Luciferase Assay Kit (Promega); digitonin and VPS34-IN1 (Sigma); puromycin (Thermo); recombinant IFN-β (R&D Systems); ELISA kits for murine IFN-α and IFN-β (PBL) and IL-6 (Biolegend).

Mouse monoclonal antibodies against FLAG and β-actin (Sigma) and HA (Covance); rabbit monoclonal antibodies against phospho-TBK1, TBK1 and SNX8 (Abcam); MITA, VPS34, IRF3, phospho-IRF3, phospho-IκBα and phosphor-MITA (Ser366) (Cell Signaling Technology), rabbit polyclonal antibody against ERGIC-53/p58 (Sigma); Alexa Fluor 555 Mouse anti GM130 (BD Biosciences) were purchased from the indicated companies. Rabbit and mouse anti-SNX8 sera were raised against a recombinant human SNX8 protein.

HEK293 cells were provided by Dr. Gary Johnson (National Jewish Center). THP-1 cells and HeLa cells were purchased from ATCC. HFFs were provided by Dr. Minhua Luo (Wuhan Institute of Virology). HSV-1 (KOS strain) (China Center for Type Culture Collection, Wuhan, China), vaccinia virus (Tian-Tan Strain) (China Center for Type Culture Collection, Wuhan, China) and ECTV (Han-Zhong Wang, Wuhan Institute of Virology, Wuhan, China) viruses have been obtained from the indicated resources.

### Constructs

Mammalian expression plasmids for HA- or FLAG-tagged VPS34 were constructed by standard molecular biology techniques. The other expression and reporter plasmids were previously described [16,19–22].

### Transfection and reporter assays

HEK 293 cells were seeded in 24-well dishes and transfected the following day by standard calcium phosphate precipitation method. HeLa cells were transfected by FuGENE. HFFs, MLFs and MEFs were transfected by Lipofectamine 2000. To normalize for transfection efficiency, pRL-TK (*Renilla* luciferase) reporter plasmid was added to each transfection. Luciferase assays were performed using a dual specific luciferase assay kit. Firefly luciferase activities were normalized on the basis of *Renilla* luciferase activities. All reporter assays were repeated for at least three times. Data shown were average values ± SD from one representative experiment.

### Real-time PCR

Total RNA was isolated from cells using TRIzol reagent (TAKARA), reverse transcribed, and subjected to real-time PCR analysis to measure mRNA levels of the tested genes. Data shown are the relative abundance of the indicated mRNA normalized to that of *GAPDH*. Gene-specific primer sequences were as follows:

*GAPDH*: GACAAGCTTCCCGTTCTCAG (forward) and GAGTCAACGGATTTGGTGGT (reverse);
*IFNB1*: *TGACTATGGTCCAGGCACAG* (forward) and TTGTTGAGAACCTCCTGGCT (reverse);
*IL6*: TTCTCCACAAGCGCCTTCGGTC (forward) and TCTGTGTGGGGCGGCTACATCT (reverse);
*ISG56*: *TCATCAGGTCAAGGATAGTC* (forward) and CCACACTGTATTTGGTGTCTAGG (reverse);
*RIG-I*: ACGCAGCCTGCAAGCCTTCC (forward) and TGTGGCAGCCTCCATTGGGC (reverse);
*CXCL10*: GGTGAGAAGAGATGTCTGAATCC (forward) and GTCCATCCTTGGAAGCACTGCA (reverse);
*TGF-*β: TACCTGAACCCGTGTTGCTCTC (forward) and
GTTGCTGAGGTATCGCCAGGAA (reverse);
*Gapdh*: ACGGCCGCATCTTCTTGTGCA (forward) and ACGGCCAAATCCGTTCACACC (reverse);
*Ifnb1*: TCCTGCTGTGCTTCTCCACCACA (forward) and AAGTCCGCCCTGTAGGTGAGGTT (reverse);
*Cxcl10*: ATCATCCCTGCGAGCCTATCCT (forward) and GACCTTTTTTGGCTAAACGCTTTC (reverse);
*Isg54*: CGAACTACCGTCTGGATGACTG (forward) and CTTCAACCAGCGCCATTGCTTG (reverse);
*Isg56*: ACAGCAACCATGGGAGAGAATGCTG (forward) and ACGTAGGCCAGGAGGTTGTGCAT (reverse);
*Rig-i*: AGCCAAGGATGTCTCCGAGGAA (forward) and ACACTGAGCACGCTTTGTGGAC (reverse);
*Il6*: TCTGCAAGAGACTTCCATCCAGTTGC (forward) and AGCCTCCGACTTGTGAAGTGGT (reverse);
HSV-1 *ICP22*: TGTTTGGAGACCAGACGGTA (forward) and
CATCGGAGATTTCATCATCG (reverse);
HSV-1 *ICP27*: GGCCTGATCGAAATCCTAGA (forward) and
GTCAACTCGCAGACACGACT (reverse).

### Co-immunoprecipitation and immunoblot analysis

Cells were lysed in 1 ml NP40 lysis buffer (20 mM Tris-HCl, 150 mM NaCl, 1 mM EDTA, 1% Nonidet P-40, 10 μg/ml aprotinin, 10 μg/ml leupetin, and 1mM phenylmethylsufonyl fluoride). For each immunoprecipitation, 0.4 ml aliquot of lysate was incubated with 0.5–2 μg of the indicated antibody or control IgG and 25 μl of a 1:1 slurry of Protein-G sepharose (GE healthcare) for at least 2 hr. The sepharose beads were washed three times with 1 ml of lysis buffer containing 500 mM NaCl. The precipitates were fractionated on SDS-PAGE, and immunoblot analysis were performed as described [23,24].

### RNAi

Double-stranded oligonucleotides corresponding to the target sequences were cloned into the pSuper-Retro RNAi plasmid (Oligoengine). The following sequences were targeted for human SNX8 cDNA: #1 (5′-CGGCAGATCTTCTCATATT-3′) and #2 (5′-CGGCAGATCTTCTCATATT-3′).

Human VPS34 cDNA: #1 (5′-GATCTGAAACCCAATGCTG-3′) and #2 (5′-GGAATGTGAAGATCAAGAT-3′).

Mouse Vps34 cDNA: (5′- GCTGTCCTAGAAGATCCCA-3′)

### RNAi-transduced THP-1 Cells

HEK293 cells were transfected with two packaging plasmids, pGag-Pol (10 μg) and pVSV-G (3 μg), and control or VPS34-RNAi retroviral plasmid (10 μg) by calcium phosphate precipitation. The cells were washed 12 hr after transfection and new medium without antibiotics was added for additional 24 hr. The recombinant virus-containing medium was filtered and used to infect THP-1 cells in the presence of polybrene (4 μg/ml). The infected THP-1 cells were selected with puromycin (0.5 μg/ml) for 2 weeks before additional experiments were performed.

### CRISPR-Cas9 Knockout

Establishment of SNX8-deficient HeLa cells and HFFs were performed as described [16]. Briefly, double-stranded oligonucleotides corresponding to the target sequences were cloned into the lentiCRISPR-V2 vector and cotransfected packaging plasmids into HEK293 cells. Lentiviral particles were collected and used to transduce HeLa cells and HFFs. The infected HeLa cells and HFFs were selected with puromycin (0.5 μg/ml) for 2 weeks before additional experiments were performed. The following sequences were targeted for human SNX8 cDNA: #1 (5′-GGGCAGGCACCATACGGTAG-3′) and #2 (5′-GACCTGCTGCACGATGGCCT-3′).

### ELISA

BMDMs were infected with HSV-1 for 18 hr. The culture media were collected for measurement of IFN-β and IL-6 cytokines by ELISA.

### Preparations of BMDMs, MEFs and MLFs

For preparation of BMDMs, the bone marrow cells were cultured in 10% M-CSF-containing conditional medium from L929 cells for 3–5 days. MEFs were prepared from day 12.5 embryos and cultured in Dulbecco’s modified Eagle’s medium (DMEM) supplemented with 10% FBS. Primary lung fibroblasts were isolated from approximately 4- to 6-week-old mice. Lungs were minced and digested in calcium and magnesium free HBSS containing 10 μg/ml type II collagenase (Worthington) and 20 μg/ml DNase I (Sigma-Aldrich) for 3 hours at 37°C with shaking. Cell suspensions were filtered through progressively smaller cell strainers (100 and 40 μm) and then centrifuged at 1500 rpm for 5 min. The cells were then plated in culture medium (1:1 DMEM/Ham's F-12 containing 10% FBS). After 1 hour, adherent fibroblasts were rinsed with HBSS and cultured in media.

### HSV-1 infection in mice

Mice were infected with HSV-1 i.p. The viability of the infected mice was monitored for 12 days. The mouse sera were collected at 6 hr after infection to measure cytokine production by ELISA.

### Confocal microscopy

Confocal microscopy was performed as previously described [22]. In Brief, cells were fixed with 4% paraformaldehyde for 15 min and then permeabilized in 0.2% Triton X-100 and stained with antibodies by standard protocols. The stained cells were observed with Carl Zeiss microscopy under a 60× oil objective.

### Statistical analysis

Student’s *t* test was used for statistical analysis with Microsoft Excel and GraphPad Prism Software. For the mouse survival study, Kaplan-Meier survival curves were generated and analyzed by Log-Rank test; P < 0.05 was considered significant.

## Supporting information

S1 Fig SNX8 is essential for DNA virus-triggered signaling in MLFs. (A) *Snx8*^*+/+*^ and *Snx8*^*-/-*^ MLFs (4x10^5^) were infected with VV, ECTV or HSV-1 (MOI = 1) for the indicated times before qPCR analysis.
(B) *Snx8*^*+/+*^ and *Snx8*^*-/-*^ MLFs (4x10^5^) were infected with HSV-1 (MOI = 1) for the indicated times before immunoblot analysis.
(C) *Snx8*^*+/+*^ and *Snx8*^*-/-*^ MLFs (4x10^5^) were transfected with ISD, DNA90 and HSV120 (3 μg/ml) for 3 h before qPCR analysis.
(D) *Snx8*^*-/-*^ MLFs were reconstituted with full length of SNX8 and its truncation mutants by lentiviral-mediated gene transfer. The reconstituted MLFs (4x10^5^) were infected with HSV-1 (MOI = 1) for the indicated times before qPCR analysis.
(TIF)

S2 Fig VPS34 is essential for DNA virus-triggered signaling. (A) HEK293 cells (1x10^5^) were firstly transfected with VPS34-RNAi (0.4 μg) for 24 h, then re-transfected with ISRE reporter (0.05 μg) and the indicated expression plasmids (0.1 μg each) for 24 h before luciferase assay.
(B) MLFs (4x10^5^) were treated with the indicated doses of VPS34 inhibitor (VPS34-IN1) for 10 h and then infected with HSV-1 (MOI = 1) for the indicated times before qPCR analysis.
(TIF)
