# Supplementary figures and images for: SNX8 modulates innate immune response to DNA virus by mediating trafficking and activation of MITA
Source: PLoS Pathog. 2018 Oct 15;14(10):e1007336. doi: 10.1371/journal.ppat.1007336 (PMC6188873; doi:10.1371/journal.ppat.1007336)

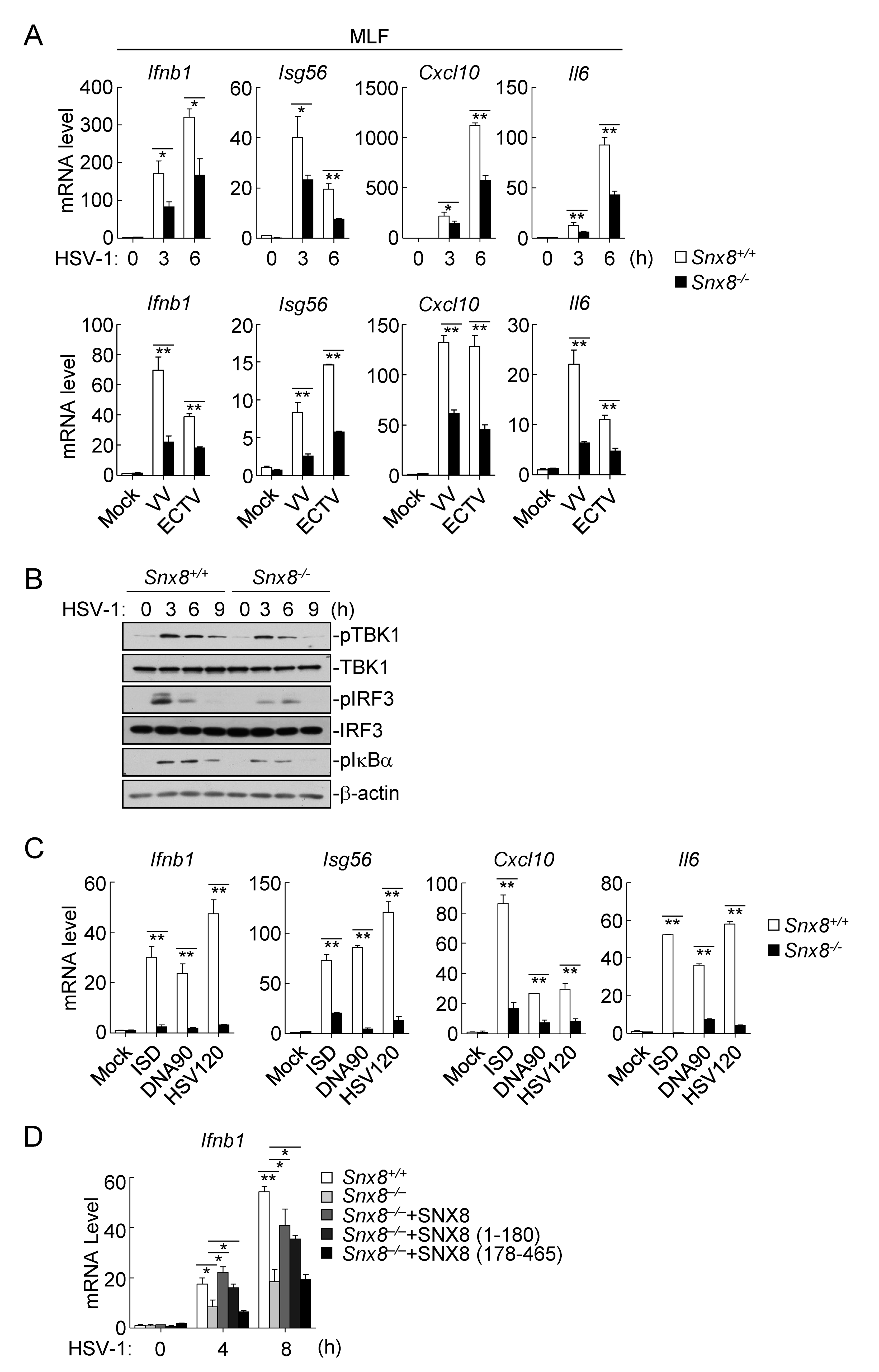

Supplement: S1 Fig — (A) Snx8+/+ and Snx8-/- MLFs (4x10^5^) were infected with VV, ECTV or HSV-1 (MOI = 1) for the indicated times before qPCR analysis. (B) Snx8+/+ and Snx8-/- MLFs (4x10^5^) were infected with HSV-1 (MOI = 1) for the indicated times before immunoblot analysis. (C) Snx8+/+ and Snx8-/- MLFs (4x10^5^) were transfected with ISD, DNA90 and HSV120 (3 μg/ml) for 3 h before qPCR analysis. (D) Snx8-/- MLFs were reconstituted with full length of SNX8 and its truncation mutants by lentiviral-mediated gene transfer. The reconstituted MLFs (4x10^5^) were infected with HSV-1 (MOI = 1) for the indicated times before qPCR analysis. (TIF) [file ppat.1007336.s001.tif]

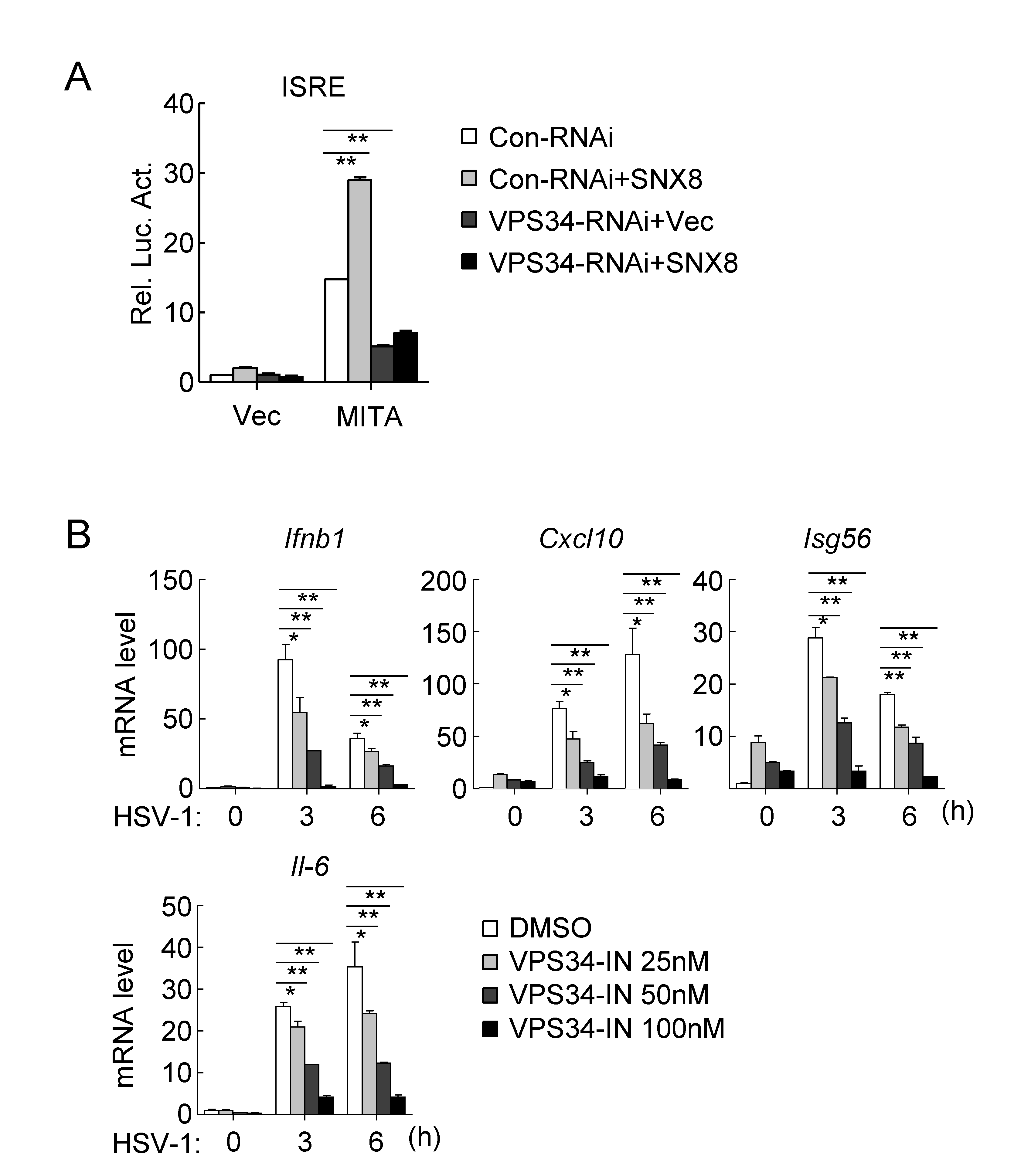

Supplement: S2 Fig — (A) HEK293 cells (1x10^5^) were firstly transfected with VPS34-RNAi (0.4 μg) for 24 h, then re-transfected with ISRE reporter (0.05 μg) and the indicated expression plasmids (0.1 μg each) for 24 h before luciferase assay. (B) MLFs (4x10^5^) were treated with the indicated doses of VPS34 inhibitor (VPS34-IN1) for 10 h and then infected with HSV-1 (MOI = 1) for the indicated times before qPCR analysis. (TIF) [file ppat.1007336.s002.tif]
